# A single-center experience of the upright proton therapy for skull-base chordomas and chondrosarcomas: Updated results

**DOI:** 10.1016/j.ctro.2024.100814

**Published:** 2024-06-29

**Authors:** Alyona Lemaeva, Igor Gulidov, Daniil Smyk, Yuliya Agapova, Sergey Koryakin, Irina Eremina, Elena Gantsova, Timur Fatkhudinov, Andrey Kaprin, Konstantin Gordon

**Affiliations:** a A. Tsyb Medical Radiological Research Center – Branch of the National Medical Radiological Research Center, Obninsk, Russia; b Obninsk Institute for Nuclear Power Engineering, National Research Nuclear University MEPhI, Obninsk, Russia; c Medical Institution, P. Lumumba People’s Friendship University of Russia, Moscow, Russia

**Keywords:** Chordoma, Chondrosarcoma, Skull base, Proton therapy, Radiation therapy, Upright position

## Abstract

**Aim:**

To access efficacy and safety of the upright proton therapy for the skull-base chordomas and chondrosarcomas.

**Materials and methods:**

The study encompasses single-center experience of proton therapy in chordomas (CA) and chondrosarcomas (CSA) of skull-base localization. We evaluate overall survival, local control and toxicity. Tumor response was assessed in accordance with RANO criteria. Treatment-related toxicity was evaluated with the help of CTCAE v 5.0 scale.

**Results:**

Proton therapy in the upright position was utilized for 51pts (patients) with CA-CSA (40 pts with chordoma and 11pts with chondrosarcoma) at the A. Tsyb Medical Radiological Research Center in 2016–2023. Median tumor volume constituted 30 cm^3^ (IQR (interquartile range) 15–41 cm^3^). Median total dose was 70 Gy_RBE_. Median number of fractions was 35. Overall survival (OS) at 1-, 2- and 3-year rates reached 98.0 %, 88.6 % and 82.7 %, respectively. Median follow-up time was 36 months. The 1-, 2- and 3-year local control (LC) rates constituted, respectively, 98 %, 78.6 % and 66.3 %. Prior surgery showed statistically significant association with better prognosis (p = 0.023). Brainstem-to-tumor dose coverage compromise became the major pattern of LC failure (p = 0.03). The late radiation toxicity reactions included temporal lobe necrosis grade 2 in 2 pts, xerostomia grade 1 in 1pt, radiation cataract grade 2 in 1pt and persistent headache grade 2 in 4 pts. Severe late toxicity reactions were observed in 2 cases (4 %): 1 myelitis grade 3 and brainstem damage grade 5 in 1pt.

**Conclusion:**

Local control was achieved in the majority of patients receiving the scanning-beam upright proton therapy for skull-base CA-CSA. The LC rates after a surgery-radiotherapy combination treatment were higher compared with irradiation alone. Pattern of failure is mostly brainstem-tumor dose compromise. The high OS and LC rates were accompanied by low toxicity incidence. Even in complex case of the skull base CA-CSA upright proton therapy shows promising clinical outcomes.

## Highlights

• Upright proton therapy shows both safety and effectiveness for complex cases of the skull base chordomas and chondrosarcomas.
• Low 95%-dose coverage and brain stem involvement remain the leading reasons for treatment failure.

## 1 Introduction

Chordomas and chondrosarcomas of the skull base are rare tumors accounting for less than 0.2 % among intracranial neoplasms [1], [2]. Chordomas (CA) originate from notochordal remnants at certain intracranial sites including the occipital bone clivus, the petrous apex of the temporal bone and the Meckel's cave. Chondrosarcomas (CSA) are derived from mesenchymal cells that produce cartilage matrix in the embryonic neurocranium and most typically localized in the petroclival region and along the petro-occipital fissure. Both CA and CSA are characterized by slow infiltrative growth deteriorating the surrounding bone and soft tissues and highly prone to local recurrence.

The current treatment standards for these tumors involve surgical resection combined to adjuvant radiation therapy [3], [4], with both steps enormously challenged by the complex anatomy of the skull base coupled to the close proximity of vital vascular and neural entities. The surgical treatment aims at radical resection with tumor-negative surgical margins, which is hard to ensure due to the aggressive and infiltrative character of tumor growth typical of CA-CSA, affecting surrounding vascular and neural structures [5]. Accordingly, the chances of total resection for CA-CSA are about 60 % or lower [5], [6], [7], [8].

Furthermore, CA-CSA are considered radiation-resistant tumors and should be irradiated in high doses (over 70 Gy) to achieve a sustainable clinical outcome; importantly, the dose distribution should be adjusted for the target volume complexity with specific regard to associated critical structures [3], [4], [9]. The advantageous radiobiological properties of proton beams enable significant reduction of the dose to normal tissues in favor of the dose to the neoplasm, as compared with photon linear accelerators [10], [11], especially when using advanced delivery techniques epitomized by the intensity-modulated proton therapy (IMPT) [12], [13], [14]. Despite its so-far limited availability, this technique has a promising potential due to the growing number of proton treatment facilities worldwide where patients with low-accessible tumors (anatomically hindered or spread locally) receive the opportunity of custom-fit radiation treatment [15].

Here we report an updated analysis aimed at evaluation of the effectiveness and safety of the pencil-beam scanning proton therapy in patients with CA-CSA of the skull base, treated in the upright position. Vertical design of proton systems demonstrates not only economic benefits, but also various clinical advantages, such as positioning comfort for head and neck pts, increased safety for the lung tumor treatment or reduced target motion [16]. Our initial experience was described in 2021 [17].

## 2 Materials and methods

The study encompasses single-center experience of proton therapy in chordomas (CA) and chondrosarcomas (CSA) of skull-base localization, implemented at the A. Tsyb Medical Radiological Research Center. The study was approved by the local Ethics Board (Protocol #185 of December 16, 2016).

The enrollment criteria included age ≥ 18 years; Eastern Cooperative Oncology Group (ECOG) performance score ≤ 3; histologically and/or clinically verified diagnosis of CA or CSA localized in the skull-base area; signed informed consent to participate in the study.

The exclusion criteria were as follows: age younger than 18 years; pregnancy or lactation; severe comorbidities (decompensated diabetes mellitus, angina pectoris, etc.); psychiatric disorders (schizophrenia, psychosis, affective-delusional); infectious or allergic conditions likely to interfere with the implementation of the therapeutic and diagnostic measures provided for in the protocol; ECOG score below 3; uncontrolled tumor-related complications.

The primarily collected data included ECOG performance score, treatment history and the presence of brainstem involvement for each participant. All patients enrolled in the study underwent magnetic resonance imaging (MRI) of the brain with contrast enhancement in T1 and T2 modes for planning of the proton therapy prior to the commencement. The patients were provided with thermoplastic masks to immobilize the head and fix its position for the accurate delivery of the beam during sessions.

**Fig. 1:**
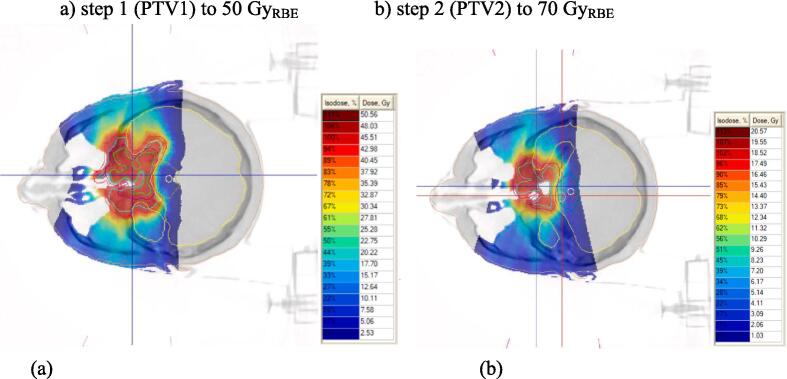
Proton treatment plan for a patient with skull-base chordoma of complex configuration after R2 resection: PTV1 (red line), PTV2 (purple line). Gross tumor volume (GTV, green contour) = 25.4 cm^3^; the numbers indicate isoeffective doses. (For interpretation of the references to colour in this figure legend, the reader is referred to the web version of this article.)

Gross tumor volume (GTV) was defined as the visible tumor volume or tumor bed. The clinical target volume (CTV) included preoperative tumor volume, residual tumor determined by computed tomography and MRI, and a margin of 10 mm, with anatomical adjustment to exclude the brainstem, optic nerves, the chiasm, intact brain tissue and other critical structures. The CTV-to-planned target volume (PTV) margin was 3 mm. The treatment was usually carried out in 2 steps with sequential narrowing of the field: (1) 50 Gy to CTV; (2) 70–74 Gy to the tumor bed or GTV. An example proton treatment plan is given in Fig. 1.

**Fig. 2:**
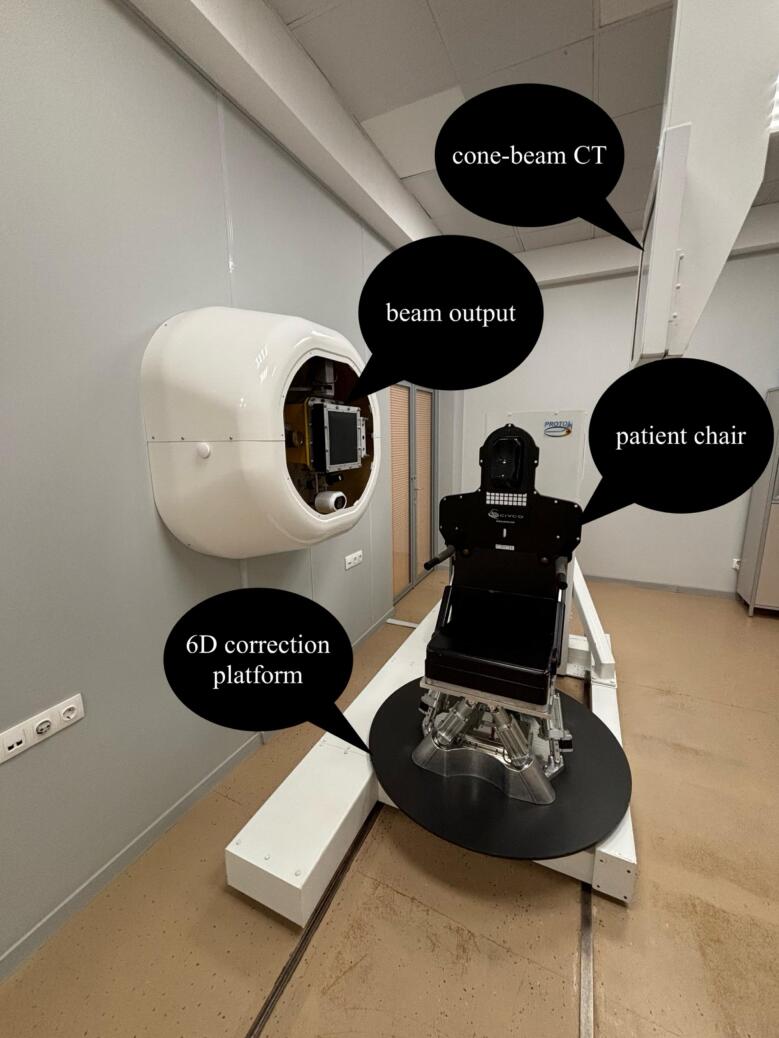
Upright patient positioning design of a medical proton complex Prometheus (JSC Protom).

The treatment was carried out with a proton therapy unit Prometheus (JSC Protom) (Fig. 2) [18]. The doses were calculated in treatment planning system ProtomTherapyPlanner ver. 1.12–2.14 using Monte Carlo algorithm (JSC Protom). We applied single-field optimized PTV-based plans, usually generated with 5–6 fields. The relative biological effectiveness (RBE) coefficient of the proton dose was accepted as 1.1. The therapy was utilized using a fixed pencil beam scanning proton technique to a patient sitting in a rotating chair [19]. The patient positioning was monitored by cone-beam computed tomography.

Restrictions prescribed for the risk organs included D_max_ 60 Gy_RBE_ for the optic nerves and chiasma, D_max_ 64 Gy_RBE_ for the brainstem and standard restrictions for other critical structures.

Clinical assessment of the tumor response was carried out using contrast-enhanced MRI of the brain once in 3 months during the 1st year follow-up and subsequently once in 6 months. The response to the treatment was assessed in accordance with the Response Assessment in Neuro-Oncology (RANO) criteria. Distant progression was assessed using chest CT each 6 months. The toxicity was evaluated using the Common Terminology Criteria for Adverse Events (CTCAE) v 5.0. Clinical outcomes calculated for the cohort included overall survival (OS), local control (LC) and toxicity incidence rates.

Statistical analysis was done in StatTech v 3.1.10 (Stattekh LLC). Survival curves were built by Kaplan-Meier method. An adjusted p-value < 0.05 indicated a statistically significant difference; p-values ≥ 0.05, but < 0.1 were noted as tendencies. The survival was analyzed by Cox regression method. Differences in overall survival and local control rates were assessed using the likelihood ratio (LR) test. The analysis of four-field contingency tables for percentages was done with Fisher's exact test. Two-group comparisons for quantitative indicators with distribution different from normal were done with Mann-Whitney *U* test.

## 3 Results

The study enrolled 51 patients (pts) including 40 pts with chordoma and 11 pts with chondrosarcoma; all tumors were localized in the skull-base area. The diagnosis was histologically verified in 48 pts. All patients received proton therapy at the A. Tsyb Medical Radiological Research Center in 2016–2023.

The patient data are listed in Table 1. Most patients (90.2 %) received the proton therapy as an adjuvant option and 5 pts (9.8 %) received it without primary surgical treatment. Most patients (74.5 %) had satisfactory ECOG score of 1. The main signs before treatment included headache, dizziness, diplopia, changes in visual acuity and ptosis. The brainstem involvement was more typical in CA (p = 0.01).

**Table 1:** Patient data.

Parameters	All M ± SD/Me	CA M ± SD/Me	CSA M ± SD/Me	P-value
Patient number	51	40	11	
Age, years	49.7 ± 15.4	51.1 ± 15.5	44.4 ± 14.3	0.195
ECOG 0	10 (19.6 %)	6	4	0.383
ECOG 1	38 (74.5 %)	31	7	
ECOG 2	2 (3.9 %)	2	0	
ECOG 3	1 (2 %)	1	0	
Brainstem involvement	33 (64.7 %)	30	3	0.010
Reirradiation	7 (13.7 %)	6	1	1.000
R0	3 (5.9 %)	2	1	
R1	3 (5.9 %)	2	1	
R2	40 (78.4 %)	31	9	
No surgical treatment	5 (9.8 %)	5	0	
Median time from surgery to radiation therapy	3 months	3 months	2 months	0.652

The proton therapy parameters are summarized in Table 2. Median GTV constituted 30 cm^3^ (IQR 15–41 cm^3^). All patients received a radical dose of proton therapy, with median total dose of 70 Gy_RBE_ (IQR 66–70 Gy_RBE_) and the median PTVs that receive 95 % of the dose reaching 78.4 % (IQR, 64.7–88.7 %). Median number of fractions (Fx) was 35 (IQR 33 – 35 Fx). One patient received combined radiation therapy for technical reasons: photon beam irradiation to a total focal dose of 40 Gy followed by proton therapy to a total focal dose of 70 Gy_RBE_. The median D_max_ received by brainstem was 59.4 Gy_RBE_ (IQR 49.5 – 63.8 Gy_RBE_), the median D_max_ delivered to the spinal cord was 23.1 Gy_RBE_ (IQR 6.6 – 46.2 Gy_RBE_).

**Table 2:** Proton therapy parameters.

Parameter	All	CA	CSA	P-value
Total dose Gy_RBE_, Me	70	70	66	0.033
V _GTV_ (_cm_^3^), Me	30	24	34	0.375
95 % dose coverage, Me	88	88	82	0.565

Reirradiation (reRT) was received by 7 pts. The median time to the reRT was 37 months (IQR 33.5 – 99.5 months). In 6 pts (11.8 %) the radiation dose was adjusted based on a history of previous RT; these patients received 46–66 Gy_RBE_ for PTV. One patient received reRT in a radical dose of 72 Gy_RBE_, considering a long gap of 15 years between the radiation therapy courses.

All patients tolerated radiation therapy well, without interruptions in the course of the treatment. The acute toxicity reactions included keratitis grade 1 in 5 pts, keratitis grade 2 in 1pt, mucositis grade 1 in 1pt and mucositis grade 2 in 3 pts.

The median follow-up after proton therapy was 3 years (range, 2–4 years).

**Fig. 3:**
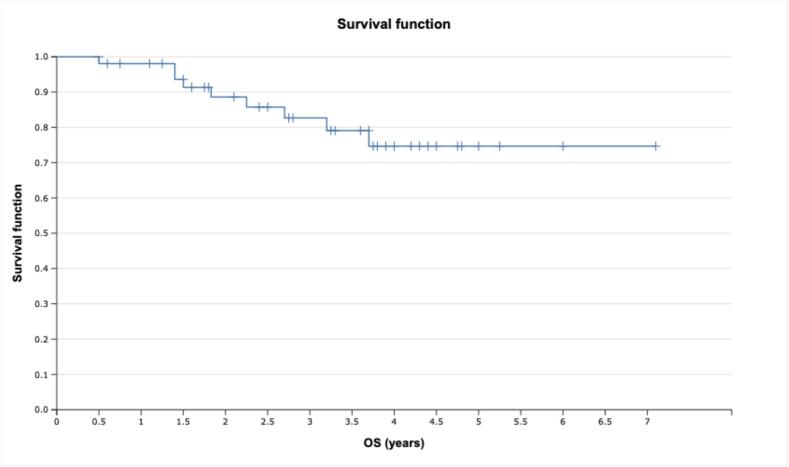
Overall survival with skull-base CA-CSA after proton therapy.

The analysis of clinical outcomes for the studied cohort of patients with skull-base CA-CSA after proton therapy indicated high OS (see Fig. 3), with 1-, 2- and 3-year rates reaching 98.0 % (95 % CI 86.9–99.7 %), 88.6 % (95 % CI 74.5–95.1 %) and 82.7 % (95 % CI 66.7– 91.5 %), respectively (see Table 3). The median survival time for the cohort was beyond the observation period.

**Table 3:** Overall survival (OS) rates for skull-base CA vs CSA after proton therapy.

Observation period, years	CSA		CA	
	OS, %	95 % CI	OS, %	95 % CI
1.0	100.0	100.0 – 100.0	97.5	83.5 – 99.6
2.0	88.9	43.3 – 98.4	88.5	72.0 – 95.6
3.0	88.9	43.3 – 98.4	81.4	62.8 – 91.3

**Fig. 4:**
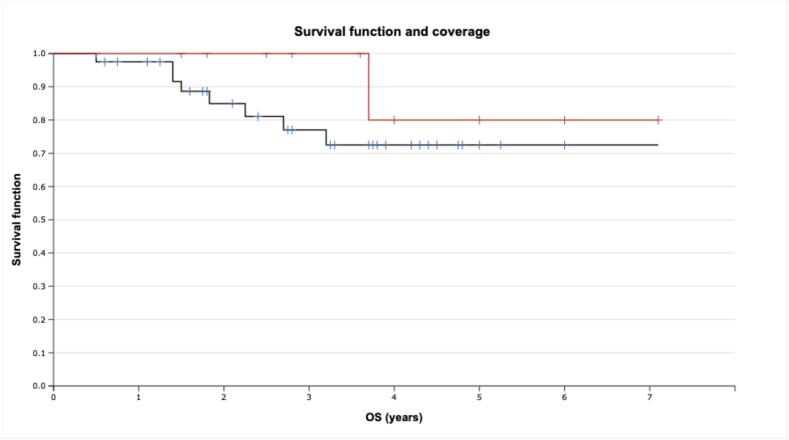
Overall survival with skull-base CA-CSA after proton therapy with and without 95% isodose coverage (LR-test). Note: red line − more than 95% coverage, blue line − less than 95% coverage. (For interpretation of the references to colour in this figure legend, the reader is referred to the web version of this article.)

The coverage of 95 % GTV with 95 % isodose was beneficial in terms of OS, with 1-year rates of 100 % vs 97.5 % for the rest of the cohort and 3-year rates of 100 % vs 77 %, respectively (see Fig. 4), albeit the trend was below significance level (p = 0.257).

The 1-, 2- and 3-year LC rates constituted, respectively, 98 % (95 % CI 86.9–99.7 %), 78.6 % (95 % CI 62.5–88.3 %) and 66.3 % (95 % CI 46.8 – 80.1 %). Specific LC rates for CA and CSA are given in Table 4.

**Table 4:** Local control (LC) rates for skull-base CA vs CSA after proton therapy.

Observation period, years	CSA		CA	
	LC, %	95 % CI	LC, %	95 % CI
1.0	100.0	100.0 – 100.0	97.5	83.5 – 99.6
2.0	74.1	28.9 – 93.0	79.9	62.3 –89.9
3.0	74.1	28.9 – 93.0	64.0	41.2 –79.9

The relapses manifested 26 ± 14 months after the therapy.

The effects of demographic data, GTV, total focal dose, histories of radiation therapy and surgery, resection volume, brainstem involvement and radiation therapy parameters on the OS and LC rates were assessed by multivariate analysis.

**Fig. 5:**
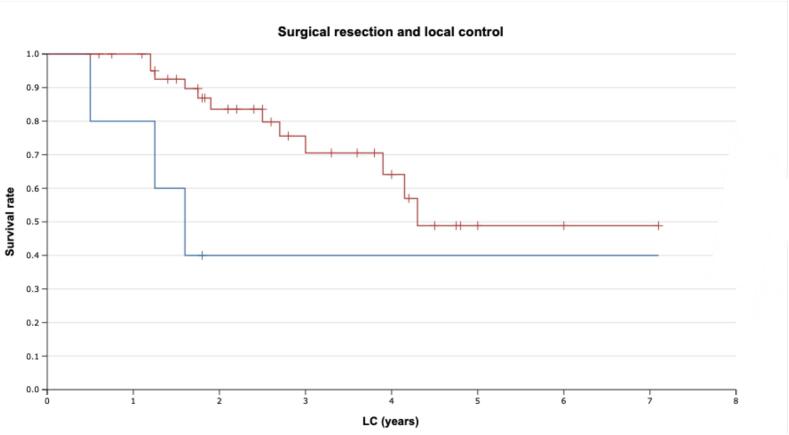
Local control in skull-base CA-CSA with and without surgical treatment prior to proton therapy. Note: red line − presence of surgical treatment, blue line − no surgical treatment. (For interpretation of the references to colour in this figure legend, the reader is referred to the web version of this article.)

The analysis of LC rates with regard to surgery using the likelihood ratio test (operated vs non-operated patients, see Fig. 5) revealed a statistically significant association (p = 0.023, HR = 0.153; 95 % CI 0.036–0.642). Other studied factors showed no significant associations with the OS and LC rates for the studied cohort.

The analysis of LC and OS rates based on the previous RT history did not show any statistically significant difference between non-irradiated and reirradiated pts (p = 0,888, HR = 0.863; 95 % CI 0.107–6.970, and p = 0,404, HR 2.027; 95 % CI 0.436–9.421, respectively).

Nine patients (17.6 %) died, six of them of non-progression-related causes. Two patients died of disease progression, one of them 27 months after the proton therapy, and 1pt died of late radiation toxicity grade 5 associated with brainstem irradiation despite only a slight overshoot of the tolerance threshold (D_max_ = 64.8 Gy_RBE_). In this case, the course was palliative, as the tumor spread massively to the brain stem and the somatic status was severe. Still, the treatment afforded two years of life for this patient. Among non-progression-related deaths, 3 pts (5.9 %) died of coronavirus pneumonia, 1pt died of gastrointestinal bleeding, 1pt died of myocardial infarction and 1pt died of stroke.

The late radiation toxicity reactions included temporal lobe necrosis grade 2 in 2 pts, xerostomia grade 1 in 1pt, bilateral radiation cataract grade 2 in 1pt and persistent headache grade 2 in 4 pts. Severe late toxicity reactions were observed in 2 cases (4 %) including myelitis grade 3 in 1pt and radiation-induced brainstem damage grade 5 in 1pt.

## 4 Discussion

A combination of surgery and adjuvant radiotherapy is considered the strategy of choice in skull-base CA-CSA. The improved clinical outcomes after irradiation were observed at total doses greater than 70 Gy_RBE_ [10], [11], [12]. The physical advantages of proton therapy enable targeted delivery of radical doses to the lesion while significantly reducing the dose to surrounding structures. The advantages of proton irradiation in skull-base CA-CSA have been acknowledged by various international guidelines [3], [4].

This study involved the active scanning intensity-modulated method (IMPT) considered the most accurate proton therapy option currently available. The analysis for a cohort of 51 pts revealed promising 1–3-year OS and LC rates. In particular, the 1- and 3-year OS constituted, respectively, 98 % and 82.7 % at corresponding LC rates of 98 % and 66.3 %, which is consistent with previously published outcomes of proton therapy for similar conditions collected at other facilities worldwide. Severe toxicity effects were observed in 2 pts only, including myelitis grade 3 in one case and post-radiation brainstem damage grade 5 in the other.

The obtained clinical outcomes confirm the previously published evidence on the effectiveness and safety of the scanning-beam proton therapy approach. In 2009, Ares *et al.* published clinical outcomes of 64 pts with skull-base CA-CSA. At median follow-up of 38 months, 5-year OS constituted 62 % for CA and 91 % for CSA, with corresponding LC rates of 81 % and 94 %, respectively. Severe late toxicity manifested in 6.25 % of the cases. The analysis identified brainstem compression (p = 0.007) and GTV over 25 ml (p = 0.03) as adverse predictors for LC [14].

The largest cohort of patients with CA-CSA after scanning-beam proton therapy (n = 222) was published in 2016. At mean GTV of 35.7 cm^3^ and mean total focal dose of 72.5 Gy_RBE_, the 5-year LC rates constituted 75.8 % for CA and 93.6 % for CSA, and the 5-year survival was 86.4 %. Adverse prognostic factors identified by the authors, affecting both LC and OS rates, included compression of the optic nerves or brainstem by the tumor, histological signs and GTV. The incidence of toxicity grade ≥ 3 was 11.3 % — somewhat higher than in other published scanning-beam proton therapy settings. The authors provide no reference guidelines for radiation burden on critical structures [20].

The multifactorial analysis carried out by us in this study revealed no significant prognostic factors apart from the history of surgical intervention for the disease. Similarly with other studies on rare tumors exemplified by skull-base CA-CSA, the power of statistical analysis was limited due to the small size of the cohort. According to published clinical findings, the effectiveness of proton therapy depends negatively on the tumor volume and brainstem involvement [20], [21].

With regard to the tumor extension, it is important to emphasize the pivotal role of combined approach in the treatment of these tumors. Here we demonstrate significantly better LC rates associated with surgical intervention prior to proton therapy. The surgical step affords a substantial reduction in the tumor volume, while smoothing the tumor shape complexity and reducing the compression of risk organs, thereby providing better opportunities for the subsequent radiation therapy.

Unlike surgery, previous RT did not show any influence on the outcomes. Obviously, a long interim before relapse (37 months in our study) levels out toxicity risks of irradiated tissues, as well as potential tumor resistance. Our own data on reRT for esthesioneuroblastoma show similar results, due to the same long gap from prior RT and high doses of irradiation. [22].

The median time from surgery was 3 months; it is noteworthy that 13.7 % of the patients had been operated several times before starting proton therapy. Since all surgical procedures were performed at external hospitals, the patient routing was complex and the data on surgical treatment were heterogeneous.

**Fig. 6:**
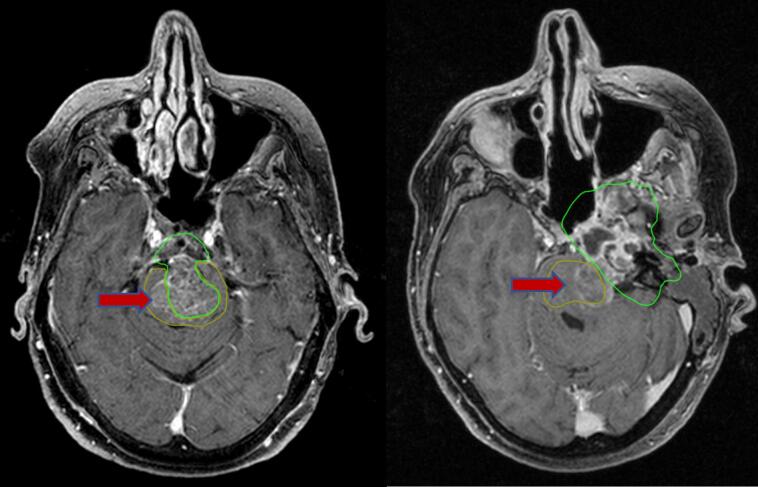
Sites of local progression after proton therapy, due to dose coverage −brainstem compromises. Note: green line shows initial tumor contour, yellow line shows initial brainstem contour, red arrow indicates recurrent loci. (For interpretation of the references to colour in this figure legend, the reader is referred to the web version of this article.)

**Fig. 7:**
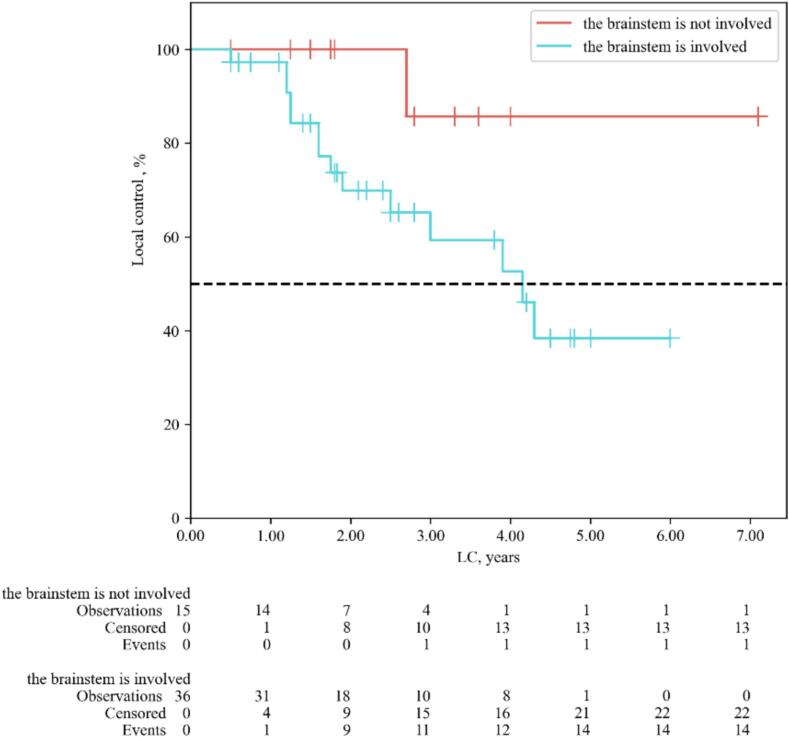
Local control of patients with skull-base CA, CSA depending on the involvement of the brain stem (LR-test).

**Fig. 8:**
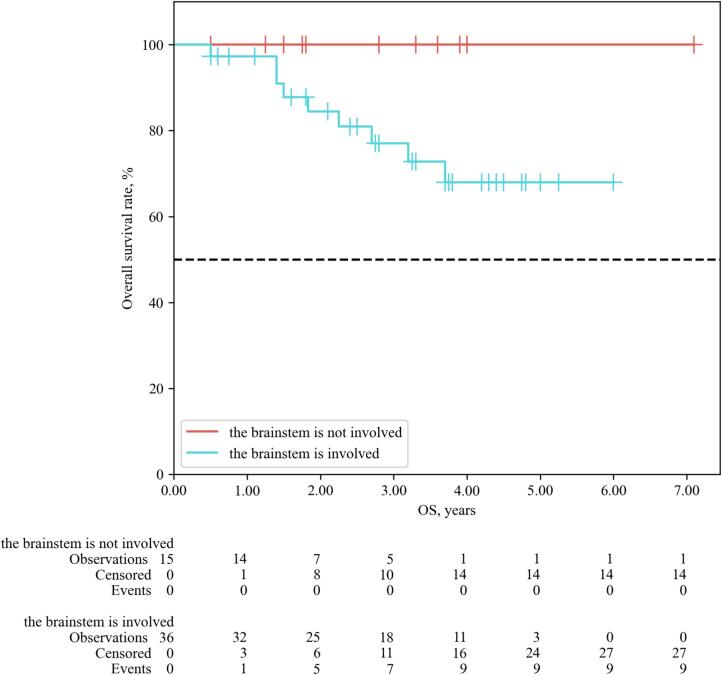
Overall survival of patients with skull-base CA, CSA depending on the involvement of the brain stem (LR-test).

According to MRI data from patients experiencing continued tumor growth after proton therapy, relapse was observed primarily at sites with low coverage (see Fig. 6). Specifically, among 15 patients with post-irradiation relapses, 14 patients (93.3 % of recurrent cases) had the irradiation target volume intersecting with the brainstem. Patients with brainstem involvement demonstrated lower LC and OS (p = 0.03 and p = 0.022, respectively) (see Fig. 7, Fig. 8). As the tolerance threshold of critical structures is lower than the required dose for the target volume, achieving optimal coverage in proximity to organs at risk, notably the brainstem, is sometimes unattainable. In general, maintaining a distance of ≥ 3–4 mm from the planning target volume (PTV) to critical structures should be ensured to reduce the risk of radiation-induced injury. The priority of reduced coverage in proximity to organs at risk has been suggested by several studies [20], [23], [24], [25]. Radiation-induced brainstem necrosis is a late, life-threatening complication that significantly affects patients with long life expectancy, such as those with cranial base tumors. There is no universal solution to address the conflict between therapy effectiveness and safety. Treatment plans, goals, and limitations are usually discussed between patients and physicians, with priorities considered on an individual basis. Despite its advantages, proton therapy introduces planning uncertainties due to high linear energy transfer areas [26]. Even with a tolerant dose map in our group, the absence of adverse events was not guaranteed (one patient experienced grade 5 brainstem injury). Further dosimetric studies, such as proton arcs or flash therapy, show promise but require extensive investigation and are still far from routine clinical use [27], [28]. These directions show promise but still require extensive investigation and are not yet ready for routine clinical use.

The histological distinction between CA and CSA is relevant for the prognosis. In CSA, high LC and OS rates can be achieved even in patients with large tumors, whereas the radiation therapy effectiveness for CA tends to be lower. Indeed, the LC and OS rates for CSA were slightly better than for CA. Advanced morphological or molecular stratification of both CA and CSA, interesting in terms of treatment planning and prognosis, was clearly beyond the scope of our consideration due to the lack of such data.

Due to the anatomical location and the high RT dose necessary for skull-base CA and CSA, proton therapy has been the most appropriate radiation modality for their treatment since its invention in 1954. Moreover, PT has afforded positive clinical outcomes even for already treated and relapsed cases of skull-base tumors [29]. Upright PT, a well-known technology, almost vanished from clinical practice due to the lack of reliable means for precision patient positioning at the beginning of the proton era. Further increasing the availability of proton treatment requires the simplification of its construction, such as the replacement of the huge and expensive gantry. New advances in image guidance, patient fixation, beam delivery, etc., have given upright approach a second chance [16]. Our own clinical practice reflects a positive experience with upright PT [17], [22].

The increasingly remote modality of follow-up examinations, certainly sparing for the patients, can be regarded as a methodologically limiting general tendency: in our study, too, the majority of patients were interviewed by phone or Also, some of the patients were out of reach for extra examinations to fully assess the toxicity.

Still, and despite the methodological limitations, it is evident that the effectiveness and safety of proton therapy in our setting is comparable with the published advanced clinical experience. While the combination approach provides better outcomes, the chances of LC show negative association with the residual tumor volume and critical structures involvement. Upgrade of the outcomes recruit enhanced routing algorithms for the patients after surgery, so that they receive a straight-away post-operative course of proton therapy without adherence to observation tactics. Since our system is synchrotron-based (so ultra-high-speed dose delivery won’t be technically available), available optimization tactic lays in the implementation of proton arc planning algorithms. The rotating chair-based unit design might perfectly fit for arc dose delivery, but requires further investigations.

## 5 Conclusion

Local control (LC) was achieved in the majority of patients who received scanning-beam upright proton therapy for skull-base CA-CSA. LC rates were higher after a combination of surgery and radiotherapy compared to irradiation alone. The primary pattern of failure observed was compromise in tumor-brainstem dose. High overall survival (OS) and LC rates were accompanied by a low incidence of toxicity. Even in complex cases of skull-base CA-CSA, the upright position design of the proton unit makes the treatment accessible for clinical practice while maintaining its quality.

## Ethics statement

This study was approved by the local ethical committee and the institutional review board of A. Tsyb Medical radiological research center—branch of the National medical research radiological center of the Ministry of Health of Russia, including the collection of all informed consent. All procedures were performed following the ethical standards of the responsible committee on human experimentation and with the Helsinki Declaration of 1964, as revised in 2013.

## Funding

The authors declare financial support was received for the research, authorship, and/or publication of this article. The study was carried out with the financial support of the Ministry of Education and Science of Russia; Agreement dated 7 October 2021 No. 075-15-2021-1356 (internal number of the Agreement: 15.SIN.21.0011); (ID: RF 0951.61321X0012) and Russian Science Foundation Grant, Agreement № 24-45-00031, https://rscf.ru/project/24-45-00031/.

## CRediT authorship contribution statement

**Alyona Lemaeva:** Data curation, Formal analysis, Resources, Writing – original draft. **Igor Gulidov:** Conceptualization, Supervision, Writing – review & editing. **Daniil Smyk:** Data curation, Writing – original draft. **Yuliya Agapova:** Data curation, Investigation, Methodology, Writing – original draft. **Sergey Koryakin:** Resources, Supervision, Validation, Writing – review & editing. **Irina Eremina:** Project administration, Resources, Supervision, Writing – review & editing. **Elena Gantsova:** . **Timur Fatkhudinov:** Funding acquisition, Project administration, Resources, Writing – review & editing. **Andrey Kaprin:** Funding acquisition, Project administration, Resources, Writing – review & editing. **Konstantin Gordon:** Conceptualization, Validation, Writing – original draft, Writing – review & editing.

## Declaration of Competing Interest

The authors declare that they have no known competing financial interests or personal relationships that could have appeared to influence the work reported in this paper.

## Data Availability

The raw data supporting the conclusions of this article will be made available by the authors, without undue reservation.
